# Quantification of myocardial perfusion with self-gated cardiovascular magnetic resonance

**DOI:** 10.1186/s12968-015-0109-1

**Published:** 2015-02-12

**Authors:** Devavrat Likhite, Ganesh Adluru, Nan Hu, Chris McGann, Edward DiBella

**Affiliations:** Department of Radiology, Utah Center for Advanced Imaging Research, University of Utah, Salt Lake City, UT USA; Department of Internal Medicine, University of Utah, Salt Lake City, UT USA; Division of Cardiology, University of Utah, Salt Lake City, UT USA; Department of Bioengineering, University of Utah, Salt Lake City, UT USA

**Keywords:** Cardiovascular magnetic resonance, Myocardial perfusion, Quantitative perfusion, ECG-gating, Self-gated

## Abstract

**Background:**

Current myocardial perfusion measurements make use of an ECG-gated pulse sequence to track the uptake and washout of a gadolinium-based contrast agent. The use of a gated acquisition is a problem in situations with a poor ECG signal. Recently, an ungated perfusion acquisition was proposed but it is not known how accurately quantitative perfusion estimates can be made from such datasets that are acquired without any triggering signal.

**Methods:**

An undersampled saturation recovery radial turboFLASH pulse sequence was used in 7 subjects to acquire dynamic contrast-enhanced images during free-breathing. A single saturation pulse was followed by acquisition of 4–5 slices after a delay of ~40 msec. This was repeated without pause and without any type of gating. The same pulse sequence, with ECG-gating, was used to acquire gated data as a ground truth. An iterative spatio-temporal constrained reconstruction was used to reconstruct the undersampled images. After reconstruction, the ungated images were retrospectively binned (“self-gated”) into two cardiac phases using a region of interest based technique and deformably registered into near-systole and near-diastole. The gated and the self-gated datasets were then quantified with standard methods.

**Results:**

Regional myocardial blood flow estimates (MBFs) obtained using self-gated systole (0.64 ± 0.26 ml/min/g), self-gated diastole (0.64 ± 0.26 ml/min/g), and ECG-gated scans (0.65 ± 0.28 ml/min/g) were similar. Based on the criteria for interchangeable methods listed in the statistical analysis section, the MBF values estimated from self-gated and gated methods were not significantly different.

**Conclusion:**

The self-gated technique for quantification of regional myocardial perfusion matched ECG-gated perfusion measurements well in normal subjects at rest. Self-gated systolic perfusion values matched ECG-gated perfusion values better than did diastolic values.

**Electronic supplementary material:**

The online version of this article (doi:10.1186/s12968-015-0109-1) contains supplementary material, which is available to authorized users.

## Background

Most Cardiovascular Magnetic Resonance (CMR) uses ECG-gating to obtain images that are not blurred by cardiac motion. However, the use of ECG-gating is a problem under certain conditions. The ECG signal can be corrupted due to radiofrequency excitations and rapidly switching gradients [1-4]. Also, the magnetohydrodynamic effect (the generation of electrical fields in the conductive blood as it flows through static magnetic fields) can disrupt the ECG signal [1]. These problems tend to be more pronounced at higher field strength such as 3 T. In addition, many times it is difficult to obtain a good ECG signal because of factors such as patient size and shape and lead placement [5].

**Figure 1:**
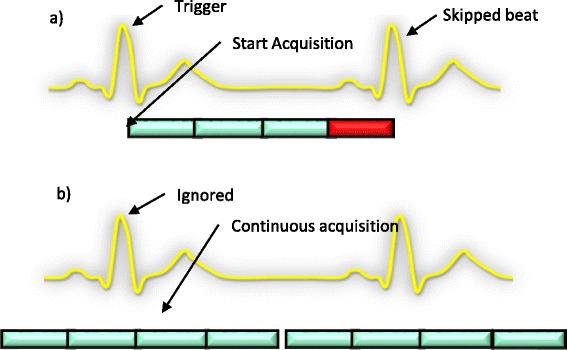
**Acquisition schematic for gated and ungated. a)** Schematic of a gated acquisition. The scanner starts acquisition with the detection of the R wave. However, even before the 4th slice is acquired, another R wave is detected. This is possible if for example the heartrate increases. **b)** Schematic of an ungated acquisition. The scanner ignores the trigger signal and does a continuous acquisition.

Various techniques, such as filtering the ECG signal or designing circuits to suppress noise pickup in ECG leads [2-4], have been proposed to obtain a good ECG-gating signal. Vector-cardiogram (VCG) based triggering, a popular method, helps to separate the electrocardiogram R-wave from the magnetohydrodynamic artifact [6]. However, even with these advances, ECG-gating is still problematic for some studies. This is evidenced in part by recent efforts towards self-gated approaches that do not require the ECG signal. The use of a separate self-gating readout [7], center of k space based gating [8] and center of mass based gating [9] have been presented recently for cine imaging at 1.5 T. Promising results for self-gated vascular imaging have been reported in [10]. Self-gated cardiac perfusion however is a bit different. Cardiac perfusion images are obtained with dynamic contrast enhanced (DCE) CMR in order to track the uptake and washout of a gadolinium-based contrast agent over time [11-14]. For cardiac perfusion DCE, T1-weighted images synchronized to the motion of the heart are obtained. The time series of images are assessed visually and/or analyzed using semi-quantitative or quantitative models to evaluate perfusion parameters. Cardiac perfusion DCE CMR acquisitions typically rely on the electrocardiogram (ECG) signal in order to fulfill the goal of synchronization. Unlike segmented acquisitions like cine imaging that acquire a portion of the k-space for each image in each heartbeat, DCE perfusion acquires all of the data each heartbeat to give a temporal resolution adequate for tracking the gadolinium contrast changes. This means that if all of the prescribed slices have not been imaged before the next R-wave trigger, then the scanner will miss acquiring during the next beat. This problem is exacerbated when slices are prescribed during rest for the stress portion of the perfusion exam. During stress imaging, the heart rate changes, so fewer slices may fit into the R-R interval. This can cause the scanner to acquire data only during alternate beats. Figure 1a shows an example where the scanner starts the acquisition process with the arrival of the first R wave. However even before the 4th slice is acquired, the second R wave arrives. Thus the second R-wave is ignored and no information is acquired during the second R-R interval. A critical missed beat, especially during uptake of contrast, can lead to erroneous quantification results.

The purpose of this study is to test if a recently proposed ungated CMR perfusion technique that does not use the ECG signal can be used to quantify myocardial perfusion in the left ventricular tissue. Initial work with this ungated acquisition for perfusion imaging has been published by our group for visual assessment of coronary artery disease [15]. The technique uses rapid undersampled readouts and compressed sensing based iterative reconstruction to acquire each image in 40–50 msec. The rapid acquisition essentially freezes the cardiac phase in each image. The data is ungated and thus not affected by the above-mentioned problems related to ECG-gating. This may also be termed a “real-time” acquisition, although the images may not reconstruct in real-time, and each slice is only acquired periodically since it has to wait for the acquisition of the other slices. The images are retrospectively classified or binned into two cardiac phases, termed near-systole and near-diastole. In this paper, the ungated data thus classified into two discrete phases is referred to as being ‘self-gated’ and the technique of classifying the data is referred to as self-gating. Unlike most self-gating methods for cine imaging, where readouts in k-space are classified into the same cardiac phase, here the self-gating operates on images. It would make less sense to classify k-space readouts together, since gadolinium contrast changes relatively rapidly and the k-space readouts from different beats would have different contrast.

In addition to qualitative assessment of coronary artery disease [15], we have presented (in abstract form) the use of self-gated perfusion data for quantification of myocardial perfusion [16,17]. A very recent paper by Chen et al. [18] supports our initial findings by reporting myocardial blood flow quantification using a self-gated approach, albeit in a single slice during breath-hold. A single slice acquisition permits much higher temporal resolution and data sharing similar to cine imaging. It remains an open question as to whether a free breathing multi-slice ungated acquisition allows for accurate quantification of myocardial perfusion. This question is studied here by comparison with an ECG-gated acquisition.

## Methods

### Overview

Several steps are involved in acquiring and processing the data. The pipeline starts with acquisition of undersampled radial k-space for approximately 1 minute following a contrast injection. The next step is reconstruction using spatio-temporal total variation (TV) constraints. For the ungated data, a rigid registration is then performed, followed by self-gating into near-systolic and near-diastolic cardiac phases. Deformable registration is then used to help suppress the residual cardiac motion in the self-gated images. Deformable registration is also used with the gated data to reduce respiratory motion. The final steps of segmenting the myocardium, generating time concentration curves and fitting to a model are the same for the self-gated and the gated datasets.

### Data acquisition

Figure 1b shows a schematic for the ungated acquisition. The scanner ignores the ECG signal and repeats the imaging sequence block of a saturation pulse and 4–5 slices continuously. A set consisting of a saturation pulse and 4–5 slices can be acquired in ~250 msec, or four times per second. Such high temporal resolution for each slice allows the time-series data to be retrospectively gated into near-systolic and near-diastolic cardiac phases.

For this study, data was acquired by scanning 7 normal volunteers (4 males, 3 females) (55.8 ± 13.2 years) in sinus rhythm at rest. A saturation recovery radial turboFLASH sequence was used for the acquisition process with TR = 2.2 ms and TE = 1.2 ms on a 3 T magnet (Verio, Siemens Healthcare, Erlangen, Germany) with voxel size 2.3 × 2.3 × 8 mm for three datasets and 1.8 × 1.8 × 8 mm for four datasets. A single saturation pulse was followed by acquisition of 4–5 slices (Two datasets with 4 slices and five datasets with 5 slices) after a delay of ~40-50 msec. Gadoteridol 0.05 mmol/kg was injected and ~230 frames were acquired over a minute with no gating and shallow breathing. Thus each slice was acquired approximately 4 times per second, regardless of heartrate. The first (basal) slice was only used for arterial input function (AIF) estimation. The next three middle slices were used for further processing.

The same gadoteridol dose and the same saturation recovery turboFLASH sequence but with ECG gating was used to acquire gated data in the volunteers. 8–10 slices were acquired in each subject. As with the ungated data, the first (basal) slice was only used for AIF estimation. The three gated slices with locations most similar to the self-gated data were processed further for comparison with the self-gated acquisition.

Prior to each of the ungated and gated acquisitions, a pre-bolus of dilute (10%) volume-matched acquisitions were performed to obtain unsaturated arterial input functions (AIFs) [19]. AIFs were also obtained from the first slice of the ungated and gated full (0.05 mmol/kg) dose acquisitions for comparison to pre-bolus AIFs.

For all of the acquisitions, the undersampled readouts were composed of 20–24 rays acquired with a starting angle offset in each time frame [20], with the angular offset repeating every 4 time frames. We have found this undersampling pattern to be equivalent to using golden ratio acquisitions for the type of reconstruction used here [21].

The order of ECG-gated or ungated acquisition was randomized, and ungated was performed first in 2 out of 7 subjects. The time between the gated and the ungated scans was 23 ± 4.7 minutes.

### Image reconstruction

The gated and ungated images were reconstructed offline using a spatio-temporally constrained reconstruction [20] with total variation (TV) constraints. The objective function *C* being minimized consists of a fidelity term and TV constraint terms [22]:

1 $$ C= argmi{n}_m{\left\Vert Em-d\right\Vert}_2^2+{\alpha}_1{\left\Vert \sqrt{\nabla_t{m}^2+\varepsilon}\right\Vert}_1+{\alpha}_2{\left\Vert \sqrt{\nabla_x{m}^2+{\nabla}_y{m}^2+\varepsilon}\right\Vert}_1 $$

The operator *E* represents the Fourier transform and the undersampling operation. The measured data is represented by *d* and the image estimate by *m*. Here *∇*_*t*_, *∇*_*x*_, *∇*_*y*_ represent the gradient operators along time, *x* and *y* dimensions respectively. *α*_1_ and *α*_2_ are empirically chosen weights for the two TV terms. *ε* is a small positive constant added to avoid numerical instability that occurs when the magnitude of the gradient is close to zero. *C* was minimized using gradient descent and the resultant images for different coils were combined using the square root of sum of squares approach. Further details of the reconstruction method are in [20].

**Figure 2:**
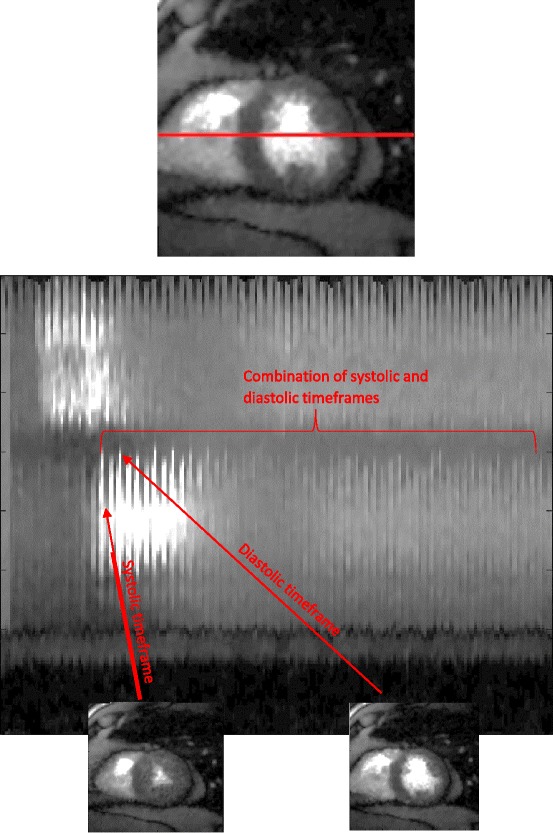
**A line profile through a single slice of an ungated acquisition, shown over time.** Systolic and diastolic timeframes are visible in the line profiles.

Figure 2 shows a line profile through one slice of a dataset acquired using the ungated technique and reconstructed as described above. The line profile shows the contrast enhancement of the RV first, then the enhancement of the LV and finally the passage of the contrast into the myocardium. The change in shape and size of the blood pool and the myocardium manifests itself in the line profile.

### Breathing motion correction

For the ungated images, a rigid registration was used first in order to reduce breathing motion. A mean image was generated by averaging proton density images, which were acquired as the first four time frames for each slice. All of the other images in the time series were rigidly registered to this single mean image automatically by using a mutual information based image registration technique. The registration was done using Advanced Normalization Tools (ANTS) [23] and setting the number of bins for mutual information based registration to 32. Each slice was processed independently.

### Self-gating

After the breathing motion correction by rigid registration, a form of self-gating was performed for the ungated data. Several self-gating methods have been compared for cardiac cine imaging [5,24], where k-space lines are grouped depending on the self-gating signal. Instead of grouping k-space lines, here we group images based on a self-gating signal. Based on comparisons between a self-gating signal from: a) correlation with a reference frame, b) the center of k-space, and c) a summed image region, the sum of all pixels in a cropped area around the heart was used to create a self-gating signal.

**Figure 3:**
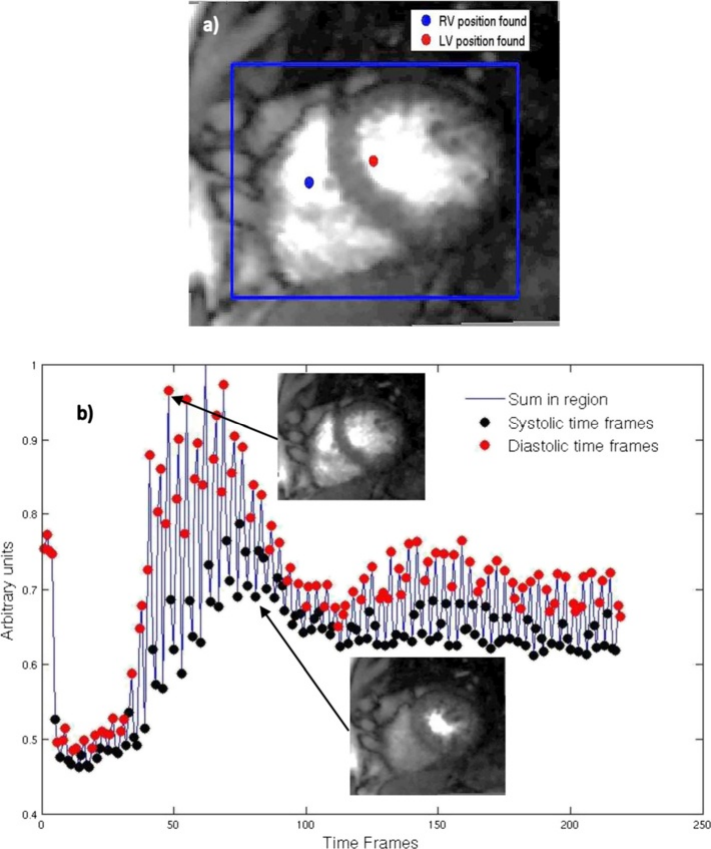
**Schematic representation of the self-gating procedure. a)** The LV and RV positions detected automatically with the rectangular ROI around the heart for self-gating. **b)** The 1D signal modulated by cardiac size change with a diastolic (peaks) and systolic (troughs) timeframe.

The cropped region around the heart was selected automatically, by first locating a point in the left ventricle (LV) and in the right ventricle (RV). The RV and LV position were found by analyzing the time to peak of the signal intensity curves from the images automatically. For this, a maximum value image was generated with the maximum value among all time-frames at each pixel. Regional clusters were then created using connected component analysis. The regional cluster with the minimum time to peak was classified as the RV and the next regional cluster to peak was classified as the LV region. Figure 3a shows an example of the LV and RV positions found along with the region around the heart. The sum of all the pixels in the region gave a 1D signal as shown by Figure 3b. The curve represents the uptake and washout of contrast agent modulated by cardiac size changes and somewhat from respiratory motion when breathing changes the content of the cropped area. The high frequency component corresponds to changes in the size of the bright LV and RV blood pools in different cardiac phases. During diastole, the blood pools are larger, and thus give the peaks in Figure 3b. Similarly, systole gives a smaller sum in the region and thus a trough in the 1D signal. An animation is provided as an additional file to make this process clear [see Additional file 1]. The animation shows an ungated dataset and the corresponding 1D signal. The timeframes corresponding to the peaks in the curve are classified as the diastolic timeframes and those corresponding to the troughs are classified as systolic timeframes. The remaining timeframes are classified as near-systolic timeframes or near-diastolic timeframes depending upon their proximity to a systolic or diastolic timeframe. All of the timeframes are used in this process. This process was automatic and resulted in two datasets, one with frames closer to systole and one with frames closer to diastole.

**Figure 4:**
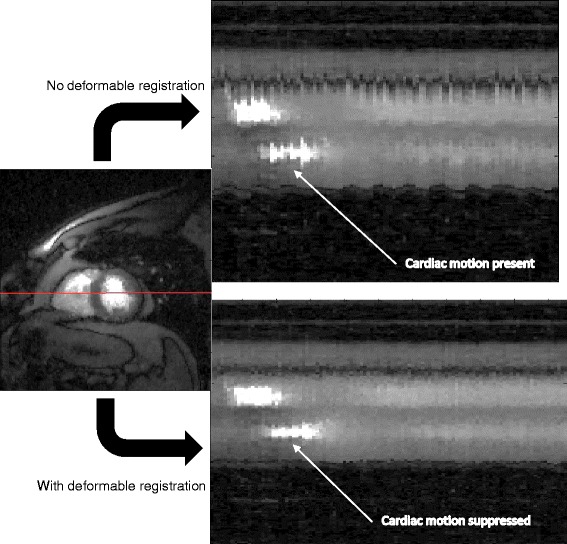
**A line profile through a self-gated systolic slice with and without deformable registration.** The suppression of the residual cardiac motion by use of deformable registration is visible.

### Deformable registration

After binning the datasets into near-systole and near-diastole, some cardiac motion was still present. Deformable registration was used to reduce the cardiac motion. This registration was complicated by the changes in contrast uptake, such that registering all of the images to a single time frame generally worked poorly. Thus a model-based deformable registration technique [25] was used instead of registering to a single frame or to neighboring time frames. The model-based deformable registration involved two steps: generation of model images, and registration of the self-gated images to these model images. The generation of model images was done by using a compartment model, as described in [26]. The model did not support motion or deformation, so these model images were “still” and acted as reference images - the self-gated images with cardiac motion were then registered to them individually at each time frame. In this way the contrast was similar between the source and target images that were being registered. The registration for each image used a symmetric image normalization method that maximized the cross-correlation within the space of diffeomorphic maps [27]. The software used for registration was the ‘Advanced Normalization Tools’ [23,28]. The sets of parameters for the deformable registration were tuned manually by testing different parameters on a few randomly chosen slices. The set of parameters selected were: Step size for transformation model = 0.25, sigma(deformation field) = 2, sigma(similarity field) = 10. For speed, the ANTs made use of a 3 level image pyramid with a maximum of 100 iterations at the coarsest resolution, 100 iterations at the next coarsest and 10 iterations at full resolution [28]. Figure 4 shows a line profile for a slice from a dataset before and after model-based deformable registration.

**Figure 5:**
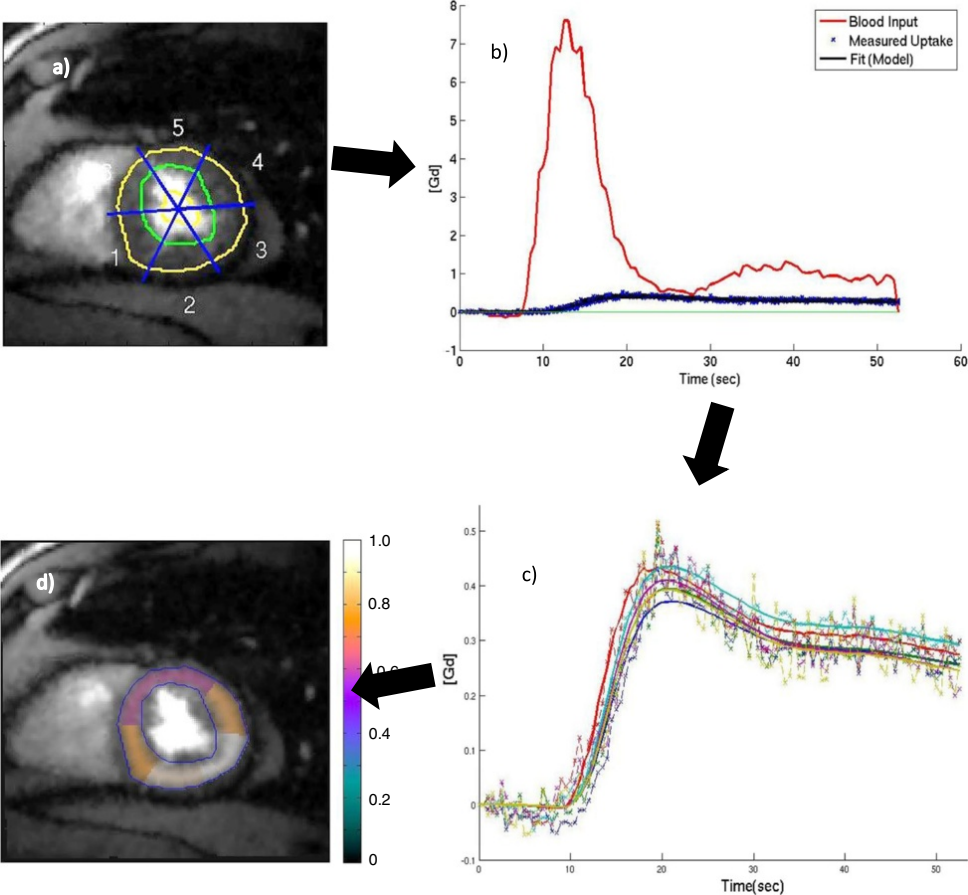
**A schematic representing the quantification process.** The various steps involved include **a)** Segmentation of the myocardium **b)** Extraction of the tissue curves and the AIF and conversion to gadolinium concentration **c)** Fitting the curves to a 2-compartment model **d)** Display of the MBF values obtained.

Deformable registration was also performed on the gated acquistions to adjust for breathing motion. The same model-based approach described above was used. For both gated and ungated, the registration was in-plane; it was assumed that there was minimal through-plane motion. A movie has been included that shows a single slice from a gated acquisition and the matching self-gated acquisition for four different subjects [see Additional file 2]. It can be seen that even though the RV moves a fair bit in a few datasets, the LV is relatively still, although some respiratory and/or cardiac motion remains.

### Quantification of perfusion

From the three motion-registered datasets (self-gated datasets at near-systole and near-diastole, and the ECG-gated dataset), extraction of contrast time curves and model fitting were peformed to generate perfusion values.

#### Tissue curves

The myocardium was segmented out manually using custom software developed in MATLAB® (The Mathworks, Inc., Natick, MA). A single time frame was segmented and its epicardial and endocardial contours were copied to the remaining images in the time series. Figure 5a shows an example of the segmented myocardium from a single self-gated (systolic) slice from the dataset pool. The segmented myocardium was divided into six circumferential regions. Average signal intensity for each region was recorded over time to give the signal intensity (*SI*) timecurves. The prescribed flip angle (α = 10°-12°) was assumed to be correct and used in the approximation eq. (2) to obtain *T1*.

2 $$ T1=\frac{-SRT}{ \log \left(\frac{M_0 \sin \left(\alpha \right)-SI}{M_0 \sin \left(\alpha \right)}\right)} $$

where *SRT* = saturation recovery time, the time from the saturation pulse to the start of the readouts for that slice. *M*_0_ is the total magnetization calculated using the proton density images acquired as the first five images at the beginning of each slice. The *T1* curves thus obtained were converted to contrast agent concentration assuming fast exchange of water [29], T1 relaxivity of 3.7 *mmol*^−1^ *sec*^−1^ for gadoteridol, and pre-contrast longitudinal relaxation time of 1660 ms for tissue at 3 T [30]. Since SRT is not clearly defined for a radial sequence that acquires the center of k space in every readout, tests were done to compare the results of conversion to Gd concentration using the time when the first ray was read and the time when the central ray was read to define SRT. The results were similar. Figure 5b shows an example of the tissue (and AIF) gadolinium concentration curves for one slice of an ungated acquisition.

#### Arterial Input Function (AIF)

In order to obtain accurate unsaturated AIFs, pre-bolus dilute (10%) volume matched acquisitions [31] were performed prior to both the gated and ungated acquisitions. The pre-bolus scan was done gated or ungated, depending on the type of scan being done next. The pre-bolus scans were reconstructed and processed in the same manner as the full dose scans. The AIF was then extracted automatically by averaging a subset of pixel values inside the endocardial border. Only values that were between 85%-95% of the maximum inside the endocardial border were included. The AIF was derived from the most basal slice (slice 1 in all cases). This AIF obtained from the low dose acquisition was converted to gadolinium concentration as described for the tissue curves in the previous section. The basal pre-bolus AIF was then scaled up by a factor of ten and used as the AIF for all of the slices of the full 0.05 mmol/kg dose acquisition.

In addition, since it is not convenient to perform the pre-bolus acquisition in future studies, the accuracy of obtaining the AIF from the first slice of the full dose acquisitions was studied. This approach was akin to a dual sequence method [32], since the saturation recovery time was relatively short (~50 msec) for the first slice of both the ungated and gated acquisitions. The AIF was obtained as in the pre-bolus scans – a region was selected automatically by considering pixels inside the endocardial border that were 85%-95% of the maximum value inside the endocardial border. This AIF was converted to Gd concentration as in eq. (2), and quantification results were generated. Since this comparison was not the main point of this study, more details and the results using this high dose AIF estimation method are given in the mini-website [see Additional file 3].

#### Model fitting

The Gd concentration tissue curves and AIF from the pre-bolus scans were fit to a compartment model [33], with a term to include blood in the tissue. The extended Kety-Tofts model is given as:

$$ {C}_t(t)={K}^{trans}{C}_b(t)*{e}^{-{k}_{ep}t}+{v}_b{C}_b(t) $$

Here *K*^*trans*^ represents the forward transfer coefficient from blood to extracellular extravascular space. *k*_*ep*_ represents the transfer coefficient from extracellular space to blood. *C*_*t*_ (*t*) and *C*_*b*_ (*t*) represent the tissue tracer concentration (tissue curves) and blood tracer concentration (arterial input function), respectively. *v*_*b*_ represents the volume of blood in the tissue region. Figure 5c shows an example of tissue curves along with their curve fits.

The *K*^*trans*^ parameter was calculated here and called myocardial blood flow (MBF). Figure 5d shows an image with the calculated MBF values overlaid. The model used is similar to the Fermi model, model independent deconvolution, or Patlak plot analysis. A previous study found that myocardial perfusion estimates using these four analysis methods were not significantly different at rest [14].

### Statistical analysis

The software package R (www.r-project.org) was used to perform statistical analysis. 7 datasets with 3 slices each and 6 circumferential regions in each slice gave a total of 126 MBF values to be compared between the methods.

Since the MBF measurements within each subject were expected to be correlated across slices and regions, a linear mixed effects model (LMEM) was used to test whether the different methods (gated, self-gated diastole, and self-gated systole) had a statistically significant effect on the mean of the MBF values. In order for two methods of measurement to be considered interchangeable, it is required that (1) there is no significant inter-method bias; (2) there is no difference in the between-subject variability of the two methods; and (3) there is no difference in the within-subject variability of the two methods [34]. We used the LMEM to test the inter-method bias and the likelihood ratio (LR) to test whether between-subject and within-subject variability were different in the gated and self-gated methods. Results with *p* < 0.05 were considered statistically significant.

A modified Bland-Altman analysis as described by Altman et al. for agreement between methods for multiple correlated observations per individuals [35] was used, along with a histogram of the measured flow values to better visualize MBF differences between the methods.

## Results

Table 1 summarizes the mean, within-subject standard deviation (SD) and between-subject SD of the MBF values by method (self-gated diastole, self-gated systole and gated). The MBF values (mean ± SD) were 0.64 ± 0.26 ml/min/g using self-gated systole, 0.64 ± 0.26 ml/min/g using self-gated diastole and 0.65 ± 0.28 ml/min/g for the gated technique.

**Table 1:** **Summary of MBF values by method**

**By method**	**Mean**	**SD**
**Self-gated systole**	**0.64**	**0.26**
Between subjects		0.23
Within subjects		0.15
**Self-gated diastole**	**0.64**	**0.26**
Between subjects		0.25
Within subjects		0.11

Table 2 reports the difference in mean MBFs between gated and self-gated diastole estimates and between gated and self-gated systole estimates. In both comparisons, the mean of the MBF values estimated using the gated method was slightly larger than those estimated using self-gated methods. On average, self-gated diastole gave a mean MBF 0.014 ml/min/g (*p* = 0.36) lower than that estimated by the gated method, and self-gated systole gave the mean of MBFs as 0.015 ml/min/g (*p* = 0.22) lower than the gated estimates. Therefore, there is no statistically significant inter-method bias when comparing gated and self-gated methods. Also, the LR tests failed to reject the null hypothesis of equal within-subject variations and equal between-subjects variations when comparing gated with self-gated diastole estimates and comparing gated with self-gated systole estimates (for all cases, *p* > 0.98). Based on the three criteria for interchangeable methods listed in the Statistical Analysis Section, the MBF values estimated from self-gated and gated methods could be used interchangeably.

**Table 2:** **Difference in mean of MBF values between self-gated diastole and gated, and between self-gated systole and gated estimated using linear mixed effects model**

**Comparisons**	**Diff. in mean of MBF**	**SE**	**p-value**	**95% conf. interval of diff.**
**Systole vs. gated**	**−0.015**	**0.012**	**0.219**	**(−0.039, 0.009)**
LR test for between subject variability			0.99	
LR test for between subject variability			0.99	
**Diastole vs. gated**	**−0.014**	**0.015**	**0.361**	**(−0.044, 0.016)**
LR test for between subject variability			1.00	
LR test for between subject variability			0.99	

**Figure 6:**
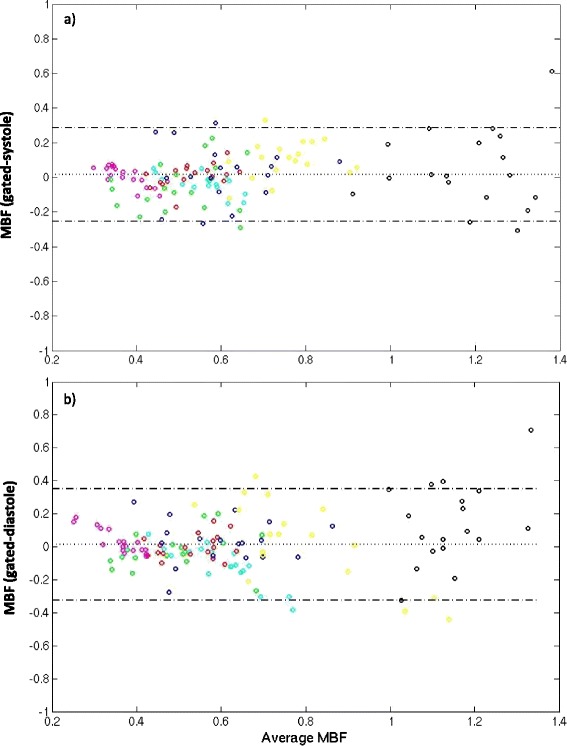
**Bland-Altman plot of the MBF from the self-gated approach and from the gated acquisition. a)** Self-gated systole with gated. **b)** Self-gated diastole with gated. The plots were created from seven datasets, three slices each with six regions in each slice.

**Figure 7:**
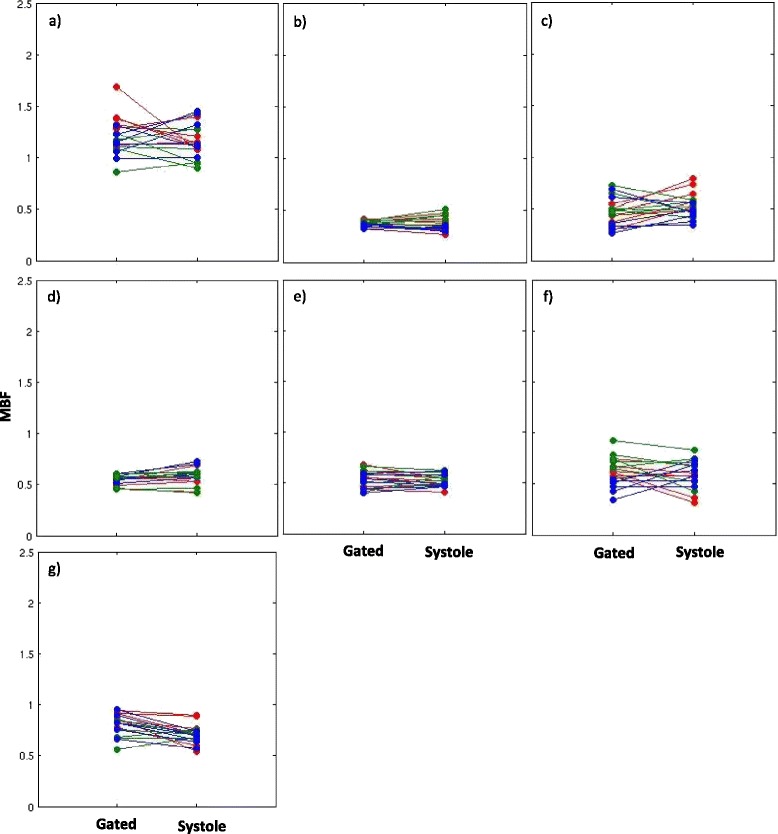
**Paired MBF plot (systole). a)-g)** Paired MBF plots for seven subjects comparing MBF obtained from gated and the self-gated systole dataset. Slices coded by color. red-basal, green-mid ventricular, blue-apical.

**Figure 8:**
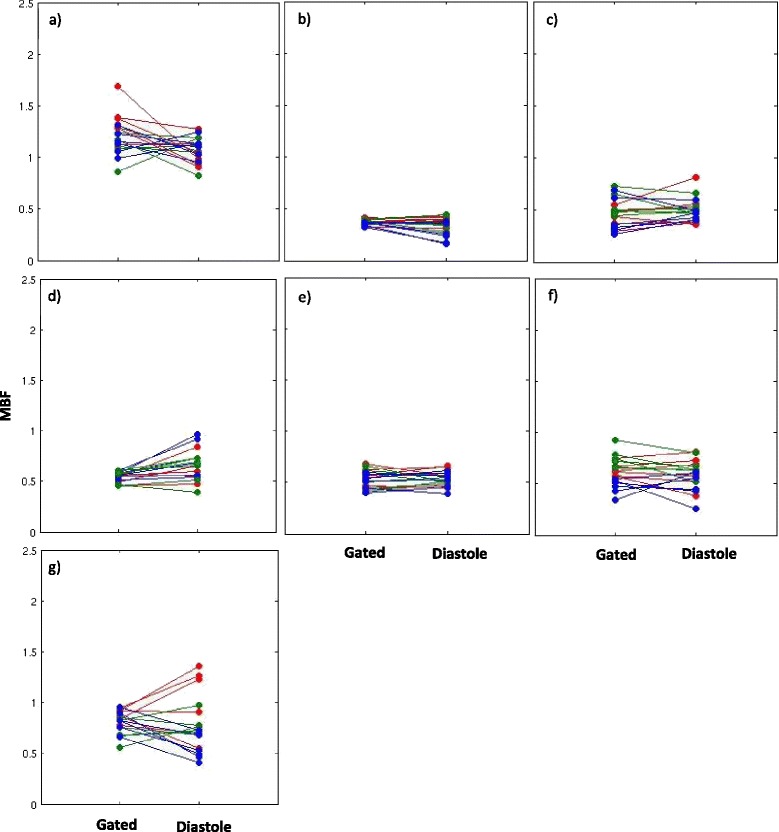
**Paired MBF plot (diastole). a)-g)** Paired MBF plots for seven subjects comparing MBF obtained from gated and the self-gated diastole dataset. Slices coded by color. red-basal, green-mid ventricular, blue-apical.

**Figure 9:**
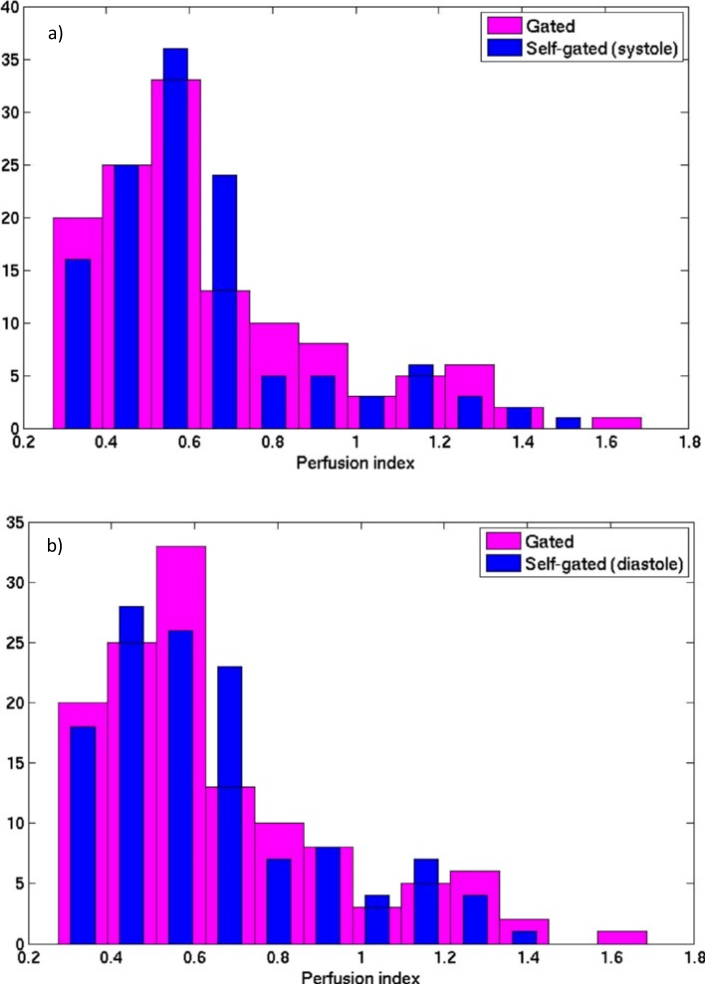
**Histogram plot showing the distribution of the MBF parameter using a) self-gated systole and b) self-gated diastole, along with the gated acquisition MBFs.** The histograms were created from the seven datasets, three slices each with six regions in each slice.

Figure 6 shows the Bland-Altman plot for the systolic and diastolic self-gated techniques. The diastolic self-gated result shows a wider spread, likely due to more challenging segmentation. Another way to look at the data is in Figure 7, which shows a paired MBF plot between the 18 MBF values (3 slices × 6 regions per slice) estimated using gated and the 18 MBF values estimated using self-gated systole for each of the seven subjects. Three different colors represent the three different slices being compared. Figure 8 shows the same type of plot for self-gated diastole. It can be seen that for each dataset the gated as well as the self-gated acquisitions give a similar mean. Figure 9 shows a distribution of the MBF from the gated acquisition and the proposed ungated acquisition for all datasets, all slices and all regions. One can see a similar distribution for both gated and the self-gated acquisition.

We also tested how well a method performed that did not use the pre-bolus for AIF estimation. As described in the Methods, quantification was done in this case using the AIF obtained from the first slice of the full dose acquisition, which had a short saturation recovery time. MBF values (mean ± std.) of 0.66 ± 0.31 ml/min/g using the self-gated systole, 0.68 ± 0.35 ml/min/g using self-gated diastole and 0.63 ± 0.30 ml/min/g for the gated technique was found. Additional results including figures for quantification using the full dose AIF can be found on the mini-website [see Additional file 3].

## Discussion

This work demonstrates the use of a new self-gated radial undersampled saturation recovery method for quantification of myocardial perfusion. This type of ungated acquisition has been shown to be useful in a preliminary study using visual qualitative analysis for diagnostic accuracy of coronary artery disease [15]. In contrast, the work here showed that an ungated acquisition followed by self-gating with deformable registration gave results similar to an ECG-gated acquisition for regional myocardial perfusion in multiple slices.

Self-gating methods have been applied to ungated cine CMR data although generally such methods “bin” k-space data instead of image-space data as is done here. Several different cine self-gating methods have been compared by Larson et al. [5]. One of these methods, the sum in the heart region, was used in this work, although it is possible that other methods could identify cardiac phase better.

### Advantages of an ungated acquisition

The use of the self-gated technique allows for quantification of perfusion without dependence on an ECG-gating signal. As well, the ungated acquisition may provide the most information during the brief passage of the contrast, in that there is very little dead time as readouts are being performed almost continuously. The use of conventional ECG-gated acquisition adds to the complexity of myocardial perfusion imaging. Even with careful setup, factors such as change in the heartrate or a missed trigger can affect the efficiency of the acquisition process. Moreover, the number of slices that can be acquired with the conventional ECG-gated acquisition is affected by the heartrate. The proposed ungated free-breathing acquisition greatly simplifies the acquisition.

### Systolic and diastolic perfusion

The splitting of the data into different cardiac phases has the additional advantage that further analysis could provide perfusion parameters as a function of cardiac phase. Other groups have studied systolic and diastolic perfusion measured with ECG-gated CMR in a single slice, and found that rest perfusion was not significantly different [36,37]. Similar results were seen here in this small study. Stress perfusion was reported to be different for systolic vs. diastolic imaging in [36,37]. Further work is needed to evaluate ungated stress perfusion quantification.

### Relation to other ungated perfusion works

In addition to our paper on qualitative self-gated perfusion [15], initial quantification results keeping only the frames that were most systolic or diastolic have been presented [16], and were followed by the use of all of the data along with deformable registration to quantify [17]. A related work [18] appeared after the initial submission of this paper. This new paper by Chen et al. [18] used the same type of self-gating approach, with the important difference of acquiring a single slice at high temporal resolution. 64 readouts were done after each saturation pulse, and then data sharing was used to obtain images every 40 msec. Only systolic frames were used to create the tissue time curves, while a set of frames with varying SRT at diastole were used to generate the AIF. Good regional agreement was shown compared to a slice from a conventional ECG-gated Cartesian acquisition in 14 subjects at rest. While a single slice could be useful for studying diffuse ischemia such as microvascular disease, it is more useful in general to obtain multi-slice quantitation. The single slice methods are not directly extendable to multi-slice, since data is not adjacent in time to be shared in the same manner. The work in this current paper shows one way to do multi-slice quantification, which used all of the acquired data along with deformable registration to give regional perfusion results similar to gated studies.

### Limitations

One limitation of the work is the number of subjects. 7 subjects at rest were imaged to provide proof of concept and help to determine how well ungated acquisitions can be quantified. Stress studies and more subjects, including those with disease, would be useful to better characterize the new approach, and will be performed as a next step.

The possibility of mismatch between the slices used for comparison between the gated and the self-gated data is another limitation of this study. However, this would be expected to only decrease the agreement between the gated and ungated scans so is not a severe limitation given the relatively close agreement of the methods.

Motion was assumed to be in-plane in both the gated and ungated studies. This is a common assumption for gated perfusion scans in the literature. Due to cardiac motion, the ungated scans are expected to have more out-of-plane motion for basal slices, which could lead to less correlation between the gated and ungated scans.

The perfusion studies were sequential with 23 ± 4.7 minutes between them, so some gadolinium contrast was present in the second scan. A number of other works have compared quantitative perfusion results after 10–20 minutes and found little effect of the remaining contrast agent. The study order was randomized here to reduce any residual effects.

While the acquisition process is unaffected by arrhythmias, the physiology of the heart, in terms of pre-load and after-load and metabolic demand, can be affected. The dynamic contrast enhanced perfusion method provides a weighted average of myocardial perfusion over ~20 seconds and so may be sensitive to load and hemodynamic changes. The subjects studied here were in sinus rhythm in order to provide a direct comparison to an ECG-gated method without concern regarding the relative effects of arrhythmias on the acquisitions.

For this study, the tunable parameters for the deformable registration process were chosen to compensate for the residual cardiac motion after binning to near-systole or near-diastole. Various parameters were experimented on test datasets and a single set that registered the test datasets well was used. The movie showing the gated and self-gated datasets [see Additional file 2] shows that there is still some motion seen in the datasets. Further improvement of the registration step is likely possible.

### Accurate arterial input function

Another important problem for quantification of MBF is the possibility of signal saturation. Studies such as [38] have analyzed the relation between contrast agent (CA) concentration and the change in signal intensity. However the dose of CA that can be used while preserving linearity of signal change depends on the acquisition. Some investigators have found that CA doses greater than 0.005 mmol/kg results in signal saturation of the AIF [39-42]. Other studies demonstrate the use of CA dose up to 0.04-0.05 mmol/kg for the quantification of myocardial perfusion with a linear relation between signal change and CA concentration [43-46]. Sequence parameters and issues regarding how linear of a response is acceptable contribute to these differences. A dose of 0.05 mmol/kg was used for all subjects in this study, with an additional low dose pre-bolus acquisition for the AIF. As well the AIFs from the lowest saturation recovery time slice were shown to be accurate [see Additional file 3], such that a pre-bolus acquisition was not necessary.

## Conclusion

A free breathing ungated perfusion acquisition self-gated into near-systole and near-diastole and using deformable registration can be used for quantification of regional myocardial perfusion. This approach is free from problems related to ECG-gating. Moreover, this technique ensures that maximum information is acquired during the brief passage of the contrast and is thus highly efficient. The simplicity of the acquisition could contribute to making quantitative CMR more accessible.

## Additional files

Additional file 1:
**Self-gating animation.** An animation showing the process of self-gating as explained in the paper. Additional file 2:
**ECG-gated and Self-gated data.** A video showing ECG-gated data and the corresponding slice of the self-gated data for four subjects. Additional file 3:
**Mini-website.** A website showing additional results obtained by using a high-dose AIF as explained in the paper.

## Abbreviations

MBF: Myocardial blood flow
ECG: Electrocardiograph
VCG: Vector-cardiogram
CMR: Cardiovascular magnetic resonance
DCE-CMR: Dynamic contrast enhanced cardiovascular magnetic resonance
AIF: Arterial input function
TV: Total variation
LV: Left ventricle
RV: Right ventricle
ANTS: Advanced normalization tools
CA: Contrast agent
SRT: Saturation recovery time
LMEM: Linear mixed effects model
LR: Likelihood ratio
