# Supplementary material for: Quantification of myocardial perfusion with self-gated cardiovascular magnetic resonance
Source: J Cardiovasc Magn Reson. 2015 Feb 12;17(1):14. doi: 10.1186/s12968-015-0109-1 (PMC4325943; doi:10.1186/s12968-015-0109-1)
Supplement: Additional file 3: — Mini-website. A website showing additional results obtained by using a high-dose AIF as explained in the paper. [file 12968_2015_109_MOESM3_ESM.zip › Webpage/index.html]

Untitled Document


**Quantification of myocardial perfusion with self-gated MRI**

Use of AIF from high dose acquisition

**I**n addition to the use of AIF using the dual bolus technique, an AIF was obtained from the first slice of the full dose acquisition. This AIF, as in case of the dual bolus acquisition was obtained automatically. This AIF however was found to be saturated. The process of conversion to Gd concentration using the expression from the article. This AIF from the first slice of the high dose acquisition was compared to the AIF obtained from the corresponding dual bolus acquisition. Results from four best datasets for gated (Fig 1a & Fig 1c) and self gated (Fig 1b & Fig 1d).

Quantification of perfuison was done using these high dose AIFs. The quantification results were compared between the gated and the self-gated acquisition as in the article.

Results:

For the 126 regions in each subject, a mean flow value of 0.66±0.31 ml/min/g using the self-gated (systole), 0.68±0.35 ml/min/g using self-gated diastole and 0.63±0.3 ml/min/g for the gated technique was found.

|  | Dual bolus | High AIF |
| --- | --- | --- |
| Gated | 0.65±0.28 ml/min/g | 0.63±0.30 ml/min/g |
| Systole | 0.63±0.26 ml/min/g | 0.66±0.31 ml/min/g |
| Diastole | 0.65±0.28 ml/min/g | 0.68±0.35 ml/min/g |


Fig 2 shows the Bland-Altman plot between the  reported by the gated and the self-gated technique. The Bland Altman plot demonstrates the absence of bias towards the gated or self-gated technique. As expected the Bland Altman plot between self-gated (diastole) and gated shows a wider spread due to difficulty in segmentation.

*Fig 3 **Left:** Bland Altman plot between gated and self-gated (systole) **Right:** Bland Altman plot between gated and self-gated (diastole)*

Fig 3 shows the histogram plots comparing the flow values obtained using the self-gated acquisition and the gated acquisitions.

*Fig 4 Histogram plot showing the distribution of flow values from **Left:** gated and the self-gated (systole) **Right:** gated and self-gated (diastole)*

Discussion:

The use of the lowest SRT slice to obtain the AIF would eliminate the need for a dual bolus acquisition thereby saving time. The proposed ungated acquisition scheme in the article provides this AIF without any seperate acquisition. Further experiments verifying the possibility of use of this lowest SRT full dose AIF need to be done before its actual use for MBF quantification.
