# Supplementary figures and images for: Quantification of myocardial perfusion with self-gated cardiovascular magnetic resonance
Source: J Cardiovasc Magn Reson. 2015 Feb 12;17(1):14. doi: 10.1186/s12968-015-0109-1 (PMC4325943; doi:10.1186/s12968-015-0109-1)

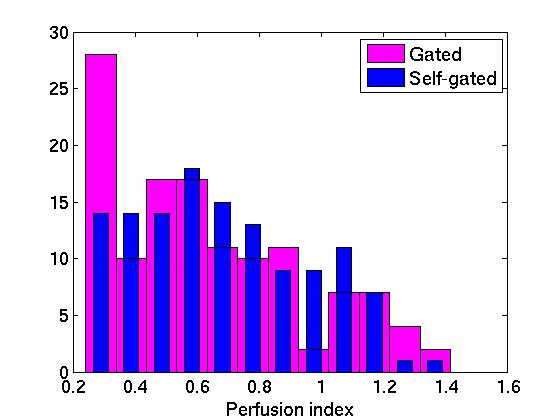

Supplement: Additional file 3: — Mini-website. A website showing additional results obtained by using a high-dose AIF as explained in the paper. [file 12968_2015_109_MOESM3_ESM.zip › Webpage/Images/hist_dias_highAIF.jpg]

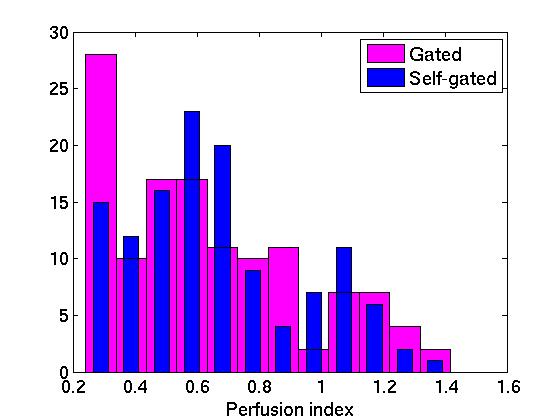

Supplement: Additional file 3: — Mini-website. A website showing additional results obtained by using a high-dose AIF as explained in the paper. [file 12968_2015_109_MOESM3_ESM.zip › Webpage/Images/hist_sys_highAIF.jpg]

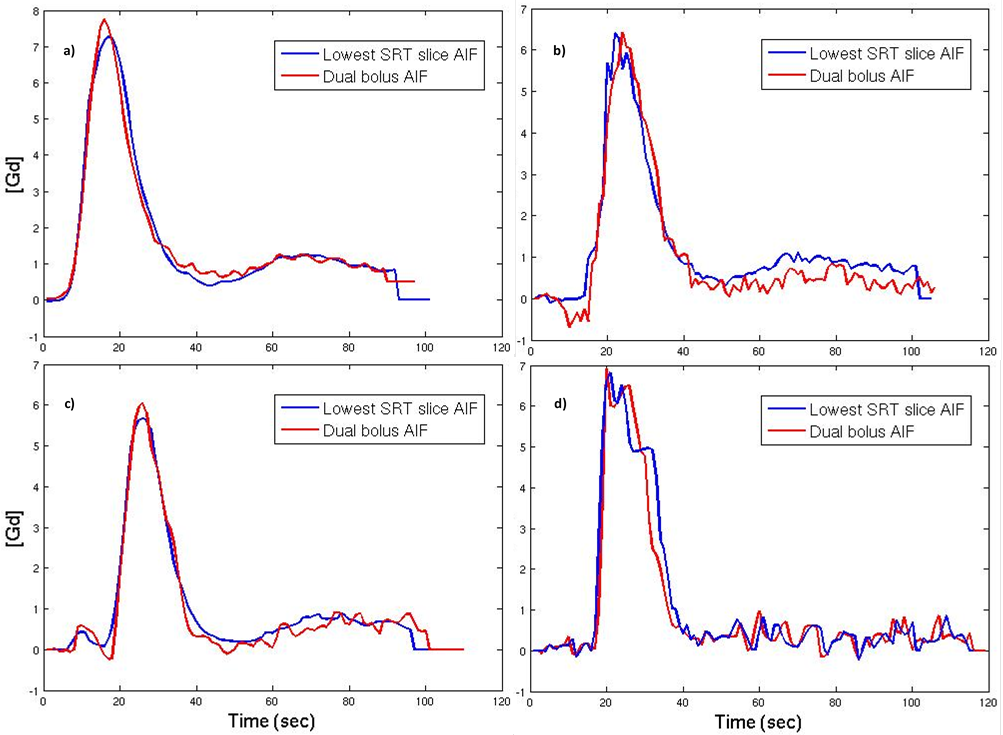

Supplement: Additional file 3: — Mini-website. A website showing additional results obtained by using a high-dose AIF as explained in the paper. [file 12968_2015_109_MOESM3_ESM.zip › Webpage/Images/image001.png]

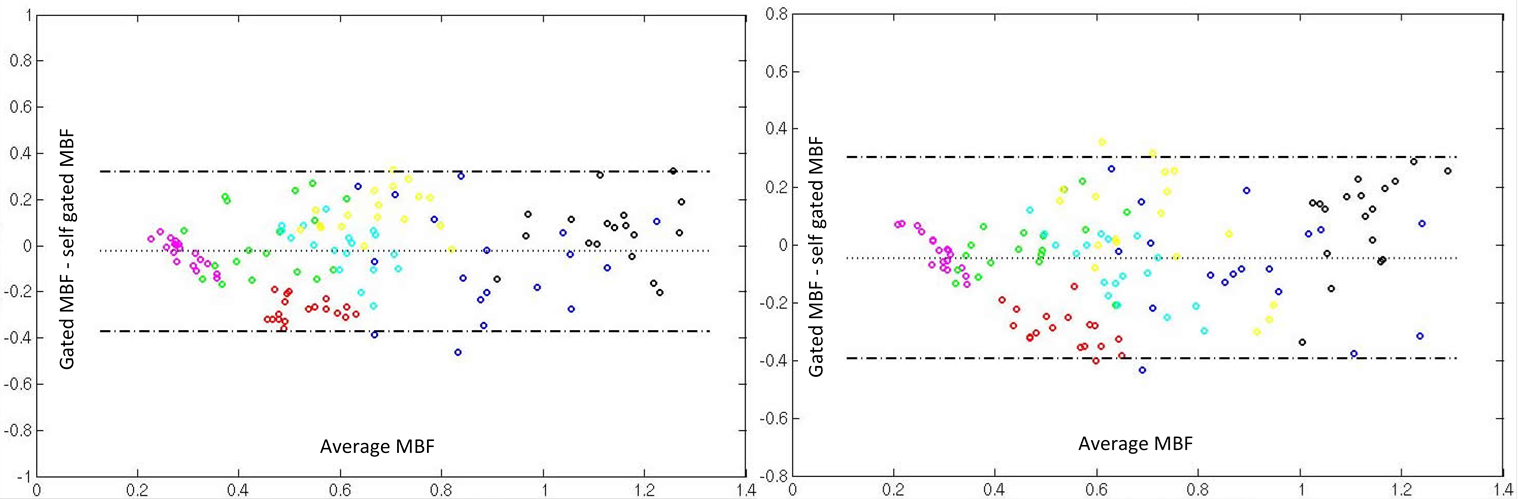

Supplement: Additional file 3: — Mini-website. A website showing additional results obtained by using a high-dose AIF as explained in the paper. [file 12968_2015_109_MOESM3_ESM.zip › Webpage/Images/image002 .png]
